# Eosinophilic myocarditis mimicking acute coronary syndrome secondary to idiopathic hypereosinophilic syndrome: a case report

**DOI:** 10.1186/1752-1947-4-40

**Published:** 2010-02-06

**Authors:** Reza Amini, Craig Nielsen

**Affiliations:** 1 Medicine Institute, Cleveland Clinic, Cleveland, Ohio, USA

## Abstract

**Introduction:**

Eosinophilic myocarditis is a rare form of myocarditis. It is characterized pathologically by diffuse or focal myocardial inflammation with eosinophilic infiltration, often in association with peripheral blood eosinophilia. We report a case of eosinophilic myocarditis secondary to hypereosinophilic syndrome.

**Case presentation:**

A 74-year-old Caucasian woman with a history of asthma, paroxysmal atrial fibrillation, stroke and coronary artery disease presented to the emergency department of our hospital with chest pain. Evaluations revealed that she had peripheral blood eosinophilia and elevated cardiac enzymes. Electrocardiographic findings were nonspecific. Her electrocardiographic finding and elevated cardiac enzymes pointed to a non-ST-elevated myocardial infarction. Echocardiogram showed a severe decrease in the left ventricular systolic function. Coronary angiogram showed nonobstructive coronary artery disease. She then underwent cardiac magnetic resonance imaging, which showed neither infiltrative myocardial diseases nor any evidence of infarction. This was followed by an endomyocardial biopsy which was consistent with eosinophilic myocarditis. Hematologic workup regarding her eosinophilia was consistent with hypereosinophilic syndrome. After being started on steroid therapy, her peripheral eosinophilia resolved and her symptoms improved. Her left ventricular ejection fraction, however, did not improve.

**Conclusion:**

Eosinophilic myocarditis can present like an acute myocardial infarction and should be considered in the differential diagnosis of acute coronary syndrome in patients with a history of allergy, asthma or acute reduction of the left ventricular function with or without peripheral eosinophilia.

## Introduction

Löffler was first to report the association between eosinophilia and heart disease in his observation of endocarditis parietalis fibroplastica and peripheral eosinophilia [1]. Regardless of the fact that eosinophilic myocarditis (EM) has been well described, due to its nonspecific clinical presentation and rapid fatal course, most of the cases are usually diagnosed on autopsy examination [2-7]. Endomyocardial biopsy remains the gold standard of diagnosis and the treatment guide in these cases.

## Case presentation

A 74-year-old Caucasian American woman presented to the emergency room of the Cleveland Clinic with a one-month history of progressive exertional chest pain. The pain was dull and diffuse. It lasted for a few minutes after exertion and was associated with shortness of breath. Physical activity made it worse and improvement was noted with sublingual nitroglycerin. She denied any nausea, vomiting, sweating, light headedness or dizziness associated with these episodes.

Her medical history was significant for long-standing asthma and hypertension. She had a stroke nine months prior to this admission in the setting of paroxysmal atrial fibrillation with near complete resolution of her neurologic deficit. Her medical history was also significant for coronary artery disease (non-ST elevation myocardial infarction) after angioplasty and stenting to the right coronary artery with a bare-metal stent; the procedure was performed four months before she presented to the emergency room. She never smoked or drank, but she did have a history of allergy to iodine.

On arrival to our emergency department her blood pressure was 105/62 mmHg, pulse was 98 beats per minute, and she was in no acute distress at rest. Her estimated central venous pressure was about 10 cm H_2_O. Her lungs revealed no wheeze or rales but had decreased breath sounds in the bilateral bases. Cardiac examination revealed a regular heart with no murmur, rubs or gallop. She had 2+ bilateral edema of the lower extremities with normal peripheral pulses.

**Figure 1:**
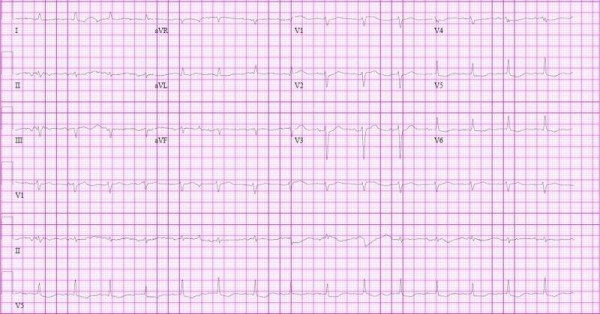
**Electrocardiogram showing low voltage, left axis deviation and questionable old anterior myocardial infarction**.

A diagnosis of non-ST elevated myocardial infarction was initially considered based on her electrocardiography, which showed sinus rhythm with low voltage, left axis deviation with ST, lateral T wave abnormalities and elevated cardiac enzymes (Figure 1). Her total creatine kinase levels peaked at 184U/L (upper limit of normal = 220U/L). Her myocardial band fraction was 54.0ng/ml or 29% (upper limit of normal = 8.8ng/ml). Her Troponin T levels peaked at 5.00ng/ml (upper limit of normal = 0.10ng/ml). Her leukocyte count was 29.59k/ul, with an eosinophil count of 18.94K/ul (64%) (upper limit of normal = 0.4k/ul).

**Figure 2:**
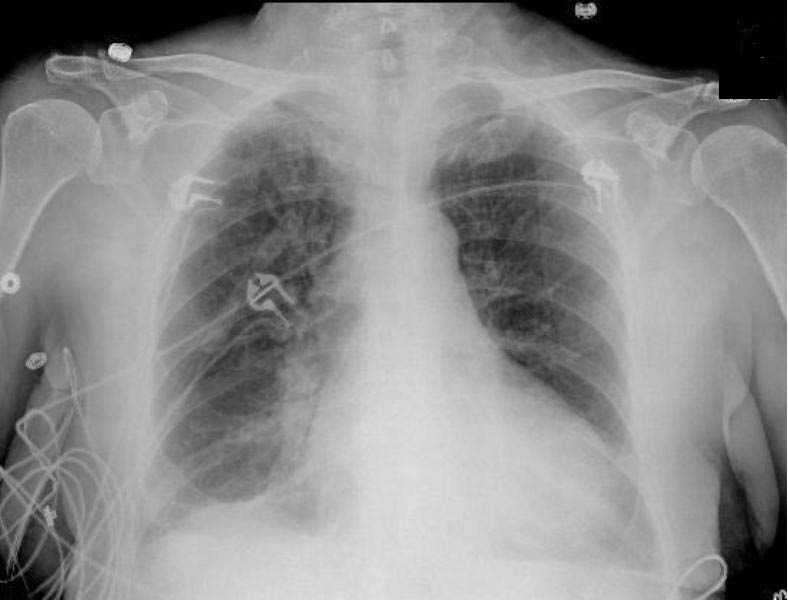
**Chest X-ray showing bilateral pleural effusion and pulmonary venous congestion**.

**Figure 3:**
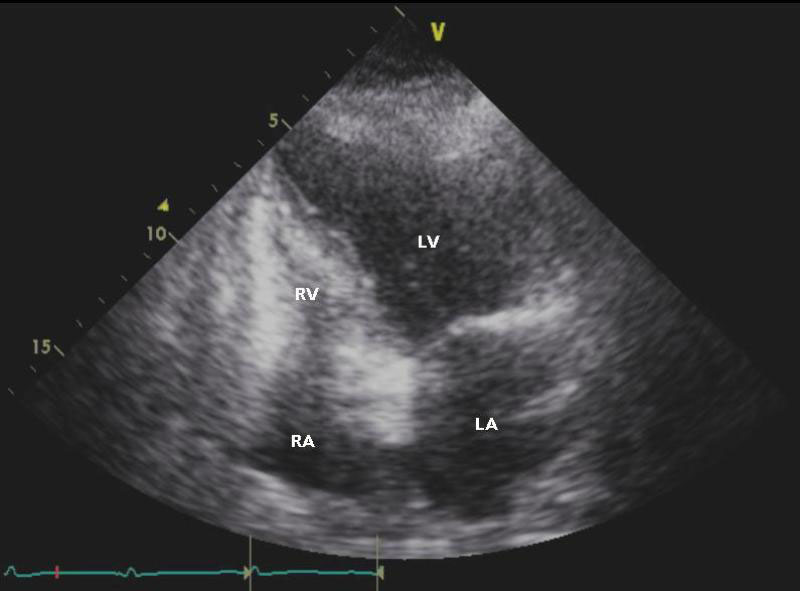
**Echocardiogram in systole (left ventricle systolic dysfunction)**.

**Figure 4:**
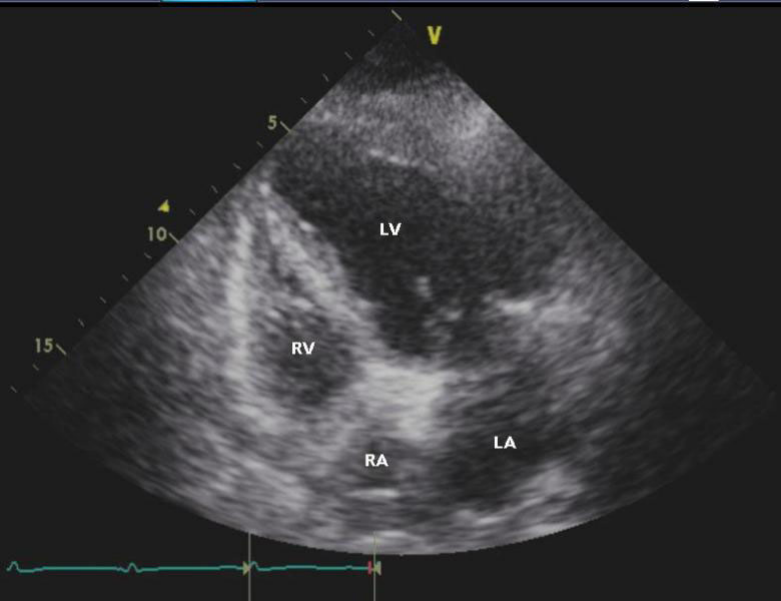
**Echocardiogram in diastole (left ventricle systolic dysfunction)**.

Chest X-ray showed the presence of cardiomegaly, bilateral pleural effusions and pulmonary venous congestion (Figure 2). Emergency echocardiography showed severe regional systolic dysfunction with an ejection fraction of 25%. The patient's left ventricular end diastolic diameter was 52 mm (Figure 3 and Figure 4). Her right ventricle was normal in size and systolic function. The aortic valve was sclerotic without aortic regurgitation and the mitral valve had 1+ regurgitation. A small pericardial effusion adjacent to the right ventricle and the right atrium was noted without signs of cardiac tamponade.

**Figure 5:**
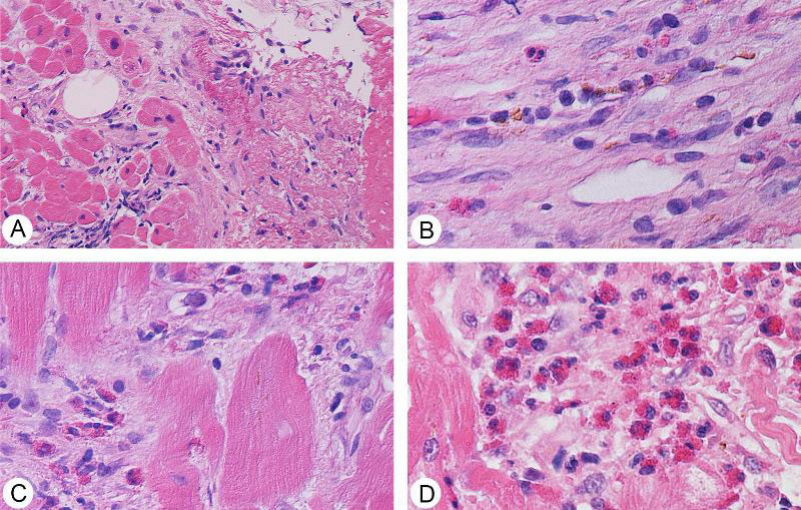
**Endomyocardial biopsy showing the following: (A) Organizing thrombus in small vessels of endocardium (Hematoxylin and Eosin staining, ×20 magnification)**. **(B) **Older areas show organized endocardial scar with rare eosinophils and hemosiderin-laden macrophages (Hematoxylin and Eosin staining, ×40 magnification). **(C) **Close-up of intact and degranulating eosinophils in the interstitial space, without myocyte necrosis (Hematoxylin and Eosin staining, ×40 magnification). **(D) **A larger cluster of non-degranulated eosinophils (Hematoxylin and Eosin staining, ×40 magnification).

Our patient was then started on aspirin, clopidogrel, statin and beta-blockers. She was also scheduled for left heart catheterization. Due to her iodine allergy, she received 1mg/kg of prednisone prior to her left heart catheterization. Her peripheral blood eosinophilia resolved after the first prophylactic treatment with prednisone. Angiography showed that our patient had a mild non-obstructive disease. She then underwent a cardiac magnetic resonance imaging, which showed a severely dilated left ventricle with severe dysfunction and multiple regional wall motion abnormalities without any evidence of infarction. On the sixth day of her hospitalization, a right ventricular endomyocardial biopsy was done, which showed endomyocardial thrombosis with eosinophilia consistent with EM. Eosinophilic infiltrate was present in the thrombosed area of the small vessels of the endocardium. The myocardium showed a repair process with lingering mononuclear cells, fibroblasts and interstitial collagen. There was no evidence of Aschoff nodules, giant cells or granulomata. A Movat stain showed no evidence of fibroelastosis. There was also no evidence of amyloid (Thioflavin-S) deposition in the interstitium (Figure 5).

Due to these findings our patient was started on a daily treatment of 70 mg of prednisone at a tapering dose. She responded well to the treatment and her chest pain resolved. The pain was presumably due to the associated pericarditis and steady decrease in her cardiac enzyme markers. An extensive workup for the cause of her eosinophilia showed negative results. This workup included negative antinuclear antibodies, negative antineutrophil cytoplasmic antibodies, negative marrow exam for malignancy, negative CHIC2 studies, negative JAK2 mutation analysis, normal serum IL-5, and negative flow cytometry for immunophenotypically abnormal T-cells associated with lymphocytic hypereosinophilic syndrome. Her stool studies and parasite serologies for strongyloides and toxocara were also negative. No vasculitis was described in any tissue specimen. The patient was therefore discharged home.

Two months later she presented to an outside facility with monomorphic ventricular tachycardia and heart failure exacerbation. She was treated, and upon discharge from this hospital she received an implantable cardioverter-defibrillator. A follow-up examination with our cardiology department five months later showed that her symptoms had improved but her ejection fraction had remained at 25%.

## Discussion

Eosinophilic myocarditis is a rare form of myocarditis [8]. It is characterized pathologically by diffuse or focal myocardial inflammation with eosinophilic infiltration, often in association with peripheral blood eosinophilia [8,9]. If this disease is left untreated, it is potentially fatal [8,10]. Eosinophilic myocarditis has been observed in 0.5% of unselected autopsy series and in more than 20% of explanted hearts from cardiac transplant recipients. The most common cause reported in these cases was related to medication [1,11]. Studies have shown that EM occurs in up to 60% of patients with hypereosinophilic syndrome [12-14].

Different etiologies have been described as a cause for EM, but the cause is frequently unknown. Well-established etiologies include hypersensitivity myocarditis due to medication (Table 1); acute necrotizing eosinophilic myocarditis (ANEM), usually with a fulminant course; hypersensitivity myocarditis associated with specific agents including smallpox, meningococcal C and hepatitis B vaccines; hypereosinophilic syndrome; Loeffler's endocarditis; tropical endomyocardial fibrosis; vasculitis such as Churg-Strauss; and malignancies including T-cell lymphoma and cancer of the lung and biliary tract [8,15].

**Table 1:** Drugs causing hypersensitivity myocarditis [20].

Drug type	Example
Antibiotic	Amphotericin B
	Ampicillin
	Chloramphenicol
	Penicillin
	Tetracycline
	Streptomycin
	Cephalosporin

Sulfonamide	Sulfadiazine
	Sulfisoxazole

Anticonvulsant	Phenindione
	Phenytoin
	Carbamazepine
	Antituberculous
	Isoniazid
	Para-aminosalicylic acid

Anti-inflammatory	Indomethacin
	Oxyphenbutazone
	Phenylbutazone

Diuretic	Acetazolamide
	Chlorthalidone
	Hydrochlorothiazide
	Spironolactone

Other	Amitriptyline
	Methyldopa
	Sulfonylurea
	Tetanus toxoid
	Dobutamine
	Digoxin
	Captopril
	Enalapril

Pathogenesis includes both immediate (immunoglobulin E degranulation of mast cells and basophiles) and delayed hypersensitivity reactions (activation of T_*H *_and IL-5 production). Eosinophilic proteins lead to increased membrane permeability in target cells by creating membrane pores that lead to cell killing [8,9]. Endomyocardial biopsy will show eosinophilic degranulation with extracellular deposition of major basic protein and eosinophilic cationic protein adjacent to thrombotic and necrotic lesions. It is not clear why eosinophils have an affinity for heart muscles [11].

Peripheral blood eosinophilia is not present in all cases, so the diagnosis of EM may not be suspected [16]. Clinical presentation is also nonspecific and has a wide spectrum. Patients may present with fever, skin rash, sinus tachycardia, chest pain, shortness of breath, symptoms of heart failure, conduction delays, and ST and T abnormalities [10,16]. Myocardial fibrosis can lead to fatal arrhythmias [10]. The diagnosis of EM is often made at autopsy. If EM is clinically suspected, an endomyocardial biopsy should be done. However, a biopsy is not very sensitive (50%) as the infiltrate is often focal [17]. If there is a high index of suspicion and the biopsy results are negative, a repeat biopsy should be performed.

The management of EM includes stopping the offending agent and starting standard treatment for heart failure. In addition, immunosuppressive therapy with a steroid, especially in patients with left ventricular failure, has been shown to improve symptoms [9,18]. In a case report by Aggarwal *et al*., a combination of azathioprine and steroids has been used to prevent the recurrence of EM [19]. In selected cases cardiac surgery (endocardectomy) and transplant have been performed [8].

## Conclusion

Our patient with EM secondary to idiopathic hypereosinophilic syndrome presented with several misleading features, including symptoms of acute coronary syndrome, nonspecific electrocardiography changes, echocardiographic findings and increased cardiac enzymes. Negative workup with regard to coronary artery disease prompted us to look for infiltrative disease with cardiac magnetic resonance imaging and endomyocardial biopsy. The endomyocardial biopsy results led to the correct diagnosis and guided the patient's treatment. In patients with a history of allergy and asthma and presenting with chest pain or symptoms of heart failure, EM should be considered. Because of the disease's potentially fatal course if left untreated, endomyocardial biopsy should be performed and repeated if necessary.

## Consent

Written informed consent was obtained from the patient for publication of this case report and any accompanying images. A copy of the written consent is available for review by the Editor-in-Chief of this journal.

## Competing interests

The authors declare that they have no competing interests.

## Authors' contributions

RA was actively involved in the management of this patient and made substantial contributions to the case report's conception and design, acquisition of data, analysis and interpretation of data. CN was involved in revising the manuscript critically for important intellectual content. All authors read and approved the final manuscript.
